# The role of managerial leadership in sickness absence in health and social care: antecedent or moderator in the association between psychosocial working conditions and register-based sickness absence? A longitudinal study based on a swedish cohort

**DOI:** 10.1186/s12889-021-12236-z

**Published:** 2021-12-04

**Authors:** Johanna Stengård, Paraskevi Peristera, Gun Johansson, Anna Nyberg

**Affiliations:** 1 Stress Research Institute, Department of Psychology, Stockholm University, SE-106 91 Stockholm, Sweden; 2 Occupational and Environmental Medicine, Region Stockholm, Stockholm, Sweden; 3 Institute of Environmental Medicine, Karolinska Institutet, Stockholm, Sweden; 4 Department of Public Health and Caring Sciences, Uppsala University, Uppsala, Sweden

**Keywords:** Leadership, Sickness absence, Psychological demands, Decision authority, Workplace violence, Longitudinal, Mediation, Moderator

## Abstract

**Background:**

The prevalence of sickness absence is particularly high among employees in health and social care, where psychosocial work stressors are pertinent. Managerial leadership is known to affect sickness absence rates, but the role leadership plays in relation to sickness absence is not fully understood; that is, whether poor leadership (i) is associated with sickness absence directly, (ii) is associated with sickness absence indirectly through the establishment of poor psychosocial working conditions, or (iii) whether good leadership rather has a buffering role in the association between work stressors and sickness absence.

**Methods:**

Four biennial waves from the Swedish Longitudinal Occupational Survey of Health (SLOSH, 2010–2016, N=2333) were used. Autoregressive cross-lagged analyses within a multilevel structural equation modelling (MSEM) framework were conducted to test hypotheses i)–iii), targeting managerial leadership, register-based sickness absence and psychosocial work stressors (high psychological demands, poor decision authority and exposure to workplace violence).

**Results:**

A direct association was found between poor leadership and sickness absence two years later, but no associations were found between leadership and the psychosocial work stressors. Finally, only in cases of poor leadership was there a statistically significant association between workplace violence and sickness absence.

**Conclusions:**

Poor managerial leadership may increase the risk of sickness absence among health and social care workers in two ways: first, directly and, second, by increasing the link between workplace violence and sickness absence.

**Supplementary Information:**

The online version contains supplementary material available at 10.1186/s12889-021-12236-z.

## Background

The prevalence of sickness absence is high among health and social care workers compared to workers in other industries in Western societies [1, 2], which is also the case in Sweden [3]. In Sweden, in 2016, nearly 30% of the employees in the health and social care industry reported suffering from psychological or physical work-caused problems [4]. It is well-established that a poor psychosocial work environment, in terms of high job demands and low job control, has an impact on health outcomes, such as depressive symptoms [5–7] and sickness absence [8–11]. During the last decades, Swedish workers in the health and social care industry reported the lowest levels of job control compared to other industries and, among women, the second-highest levels of job demands [12]. Also workplace violence has been shown to be particularly common in the healthcare industry [2, 13]—where the perpetrators are often patients and clients [14]—and to be associated with sickness absence [2, 14]. The association between managerial leadership and health outcomes among employees is also well-established [15–17], in terms of, for example, (self-reported) sickness absence [18]. With regard to the pressured healthcare sector, it is argued that leadership plays a particularly significant role in the creation of a healthy environment for workers [19]. However, the role managerial leadership plays in relation to sickness absence among health and social care workers is not fully understood; that is, whether poor leadership (a) is associated with more sickness absence directly, (b) is associated with more sickness absence indirectly through the establishment of poor psychosocial working conditions, and/or (c) whether good leadership has a buffering role in the association between psychosocial work stressors and sickness absence. In the present study we intend to investigate these different routes using a Swedish cohort study.

### Managerial leadership and its association with sickness absence

Managers can influence and shape the psychosocial work environment, as measured by established work stress models (e.g., the demand–control model by Karasek and Theorell [20, 21]) of his or her subordinates. For example, managers have influence over the amount of demands put on employees and the decision authority allowed, and also contribute to creating a secure climate for employees. The psychosocial work environment is, however, also determined by organizational factors that most first line and middle managers are part of but have little influence over.

In the present study, we define good managerial leadership as rudimentary managerial behaviours targeting the relationship with the employees, such as providing clear goals, sufficient power, feedback, and support. Poor managerial leadership, as measured in the present study, is defined as the absence of such behaviours. The leadership measure used in the present study was originally developed as part of an instrument for the assessment of workers’ stress profiles [22, 23]. This is in contrast to most leadership measures where the focus has been on performance outcomes, for example the well-established constructs of transactional and transformational leadership [24, 25].

The lack of attentive and constructive leadership, not providing clarity, employee participation, control, and support, is assumed to affect work stress as measured by high job demands and poor decision authority. For example, lack of managerial clarity and feedback may influence employee perceptions of the demands associated with their job position, and not allowing influence that corresponds to employee responsibilities, and a lack of sufficient information and goal clarity, may be associated with employee perceptions of their decision authority. In health and social care organisations, the prerequisites for a healthy psychosocial work environment is, however, often determined by political decisions far beyond middle- and first-line managers’ control. An attentive and constructive leadership could, however, buffer negative health effects of high demands and low decision authority that are inherent in the job description. A clear and attentive leadership also has potential to influence the safety in health and social care organisations, in which violence from patients and clients potentially occurs to a lesser extent. When it occurs, which is inevitable at least in some parts of the health and social care organisations, support from the manager may buffer its negative health outcomes.

In previous studies using the present managerial leadership scale, for example, a negative association with ischaemic heart disease has been shown [22]. With regard to sickness absence, subdimensions such as goal clarity and received recognition have been found to decrease sickness absence [26]. Thus, our first hypothesis is that poor managerial leadership is associated with more sickness absence.

### The mediating role of psychosocial work stressors in the association between leadership and sickness absence

A few studies have examined and found support for the theory that job demands [27–29] and job resources [29–31] have a mediating role in the association between leadership and mental ill health or psychological well-being. However, conclusions regarding the temporal order of the associations could not be drawn because most of these studies were based on cross-sectional data (or at most two time points). Moreover, few targeted the health and social care industry, and generally only self-reported data were used. To the best of our knowledge no study has been published investigating the mediating role of psychosocial work stressors in the association between managerial leadership and sickness absence with a longitudinal study design. Based on previous cross-sectional studies on mental ill health, our second hypothesis is that psychosocial work stressors, in terms of high psychological demands (2a), low decision authority (2b) and exposure to workplace violence (2c), partly mediate the association between poor managerial leadership and sickness absence.

### The moderating role of managerial leadership in the association between psychosocial work stressors and sickness absence

Scholars have argued that good leadership could be seen as a job resource that may buffer against detrimental psychosocial working conditions, such as high job demands, on health outcomes [32]. Thus, our third hypothesis is that managerial leadership moderates the association between psychosocial work stressors and sickness absence, such that good leadership buffers against poor working conditions in terms of, high psychological demands (3a), low decision authority (3b) and exposure to workplace violence (3c). Only a few studies have previously put this assumption to the test [32], and they have had mixed results [33, 34]. To the best of our knowledge, no study has been published investigating the moderating role of managerial leadership in the association between psychosocial work stressors and sickness absence.

### Aim of the present study

The aim of the present study was to examine the role of managerial leadership in the association between psychosocial work stressors (high psychological demands, low decision authority and exposure to workplace violence) and sickness absence among health and social care workers. The specific research objects were to investigate whether: (1) poorer perceived leadership of the closest manager is associated with higher risk of sickness absence, (2) psychosocial work stressors mediate the association between poor managerial leadership (of the closest manager) and sickness absence, and (3) perceived managerial leadership of the closest manager moderates the association between psychosocial work stressors and sickness absence.

## Methods

### Study population

In the present study, data from four waves (2010, 2012, 2014 and 2016) of the Swedish Longitudinal Occupational Survey of Health (SLOSH) were used. Starting in 2006, Statistics Sweden has collected data every second year by means of questionnaires, following the same respondents. In 2008 and 2014 more respondents were included. SLOSH is a largely representative cohort of the Swedish working population with the aim of examining work environment and health. For a more thorough description, see [35].

Inclusion criteria were that the respondent worked as a health or social care worker during at least two data collections. More waves were included if, on these occasions, the individual was also working in these occupations. Occupation was self-reported and then by Statistics Sweden coded into register data [36]. Thereafter, we listed occupational codes that were relevant with respect to the intended study population, that is, employees who worked as health or social care workers (e.g., a nurse, physician, care assistant, social worker, psychologist, therapist).

The final sample consisted of 2,333 individuals, of which 87.6% were women. The mean age was 49.9 (9.5) and 54.1% had a university degree. Of these, 78.4% were married/cohabitant and 51.8% had children living at home. Only associations were targeted where the individual kept his/her manager between two subsequent waves.

### Variables

All variables were measured at all four time points.

#### Managerial leadership

Managerial leadership was assessed with nine questions [22] where the participants were asked to evaluate the behaviour of the closest manager, such as whether he/she gave enough information, communicated clear goals and expectations, gave sufficient power and was good at pushing through and carrying out changes, but also whether the manager was supportive, encouraged participation, gave positive feedback and cared about the employees’ professional development. The managerial leadership scale is the leadership climate dimension of the validated Stress profile instrument [23] that was originally developed for assessing psychosocial stressors in the workplace and in private life. The instrument is based on stress research and established theories, and has been validated and tested in several workplaces in Sweden [23]. The Stress profile has been influential on, for example, the development of the well-used Copenhagen Psychosocial Questionnaire (COPSOQ) [37]. Since one question was missing in one SLOSH wave, this particular item (concerning receiving criticism from the leader if something that was not good was done) was excluded from the analyses. The response alternatives ranged from 1: “often” to 4: “never”. The Cronbach’s alpha for the remaining nine questions ranged from 0.90 to 0.91 over the SLOSH waves. Table S1 in the supplementary material presents descriptives for the nine items at the first wave. For the purpose of the moderator analyses the participants were classified as having a poor manager or having a good manager (using the median value as the cut-off).

#### Psychosocial work stressors

The measures of psychological demands and decision authority were both obtained from the Swedish version of the Demand-Control-Support-Questionnaire (DCSQ) [38, 39]. Psychological demands were assessed with five items (working fast, working intensively, too much effort, (not) enough time and conflicting demands), and decision authority was measured with two items (what to do at work and how to do the work). The response alternatives ranged from 1: “often” to 4: “never/almost never”. For psychological demands Cronbach’s alpha ranged from 0.74 to 0.78 and for decision authority from 0.74 to 0.77. Items were reversed so that higher values represented more work stressors.

#### Workplace violence

Workplace violence was measured with a single question: “Were you exposed to violence or the threat of violence in your work during the last six (twelve) months?” In 2010 the time period that was referred to in the question was the last twelve months, whereas thereafter (2012, 2014 and 2016) the last six months were referred to. The answer alternatives were dichotomised to “no” or “yes”, where yes indicated at least once during the period in question. An increase in the number of workers exposed to violence between the first and the second measurement points was observed (20.9% in 2010, 25.9% in 2012, 24.3% in 2014, 24.2% in 2016).

#### Register-based sickness absence

In Sweden, if not returning to work after seven days of sickness absence, the employee needs a doctor’s certificate. After seven additional days, the employee can receive sickness benefit (preventive sickness benefit, rehabilitation allowance and occupational injury allowance) from the Swedish Social Insurance Agency. In the present study, register-based sickness absence (net days) refers to the number of days per year of receiving sickness benefit from the agency. One register-based sickness absence day could correspond to either a full day (100%) of sickness benefit, two days of 50% or four days of 25%. We dichotomised the variable into (1) no register-based sickness absence, and (2) one or more register-based sickness absence days.

#### Covariates

Information on gender, age and educational level were obtained from register data. Information on civil status (married/cohabiting or not) and parental status (children living at home or not) was self-reported.

### Analytical strategy

Hypotheses were tested using autoregressive cross-lagged models within a multilevel structural equation modelling (MSEM) framework with both observed and latent variables. These models make it possible to address the reciprocal temporal relationships among exposure variable, mediators/moderators and outcome, and also account for the multiple levels of the data i.e. multiple measurement points (level 1-within-person level) nested within individuals (level 2-between level). More precisely we used a two-level structural equation model (SEM) that allows partitioning of between- and within-person effects to account for two inherent types of heterogeneity, within-person across time and between-person [40–42].

Before testing the hypotheses, we tested the measurement invariance in the latent variables of psychological demands, decision authority and leadership, which all showed good fit statistics and no indication of measurement variance over time.

In a first step, we examined the bivariate multilevel structural cross-lagged relationships between managerial leadership (exposure variable) and sickness absence (outcome). Managerial leadership was measured at the first time point (t-1, years 2010, 2012, and 2014) and sickness absence at the subsequent time point (t, years 2012, 2014 or 2016). The cross-lagged paths estimated the effect of one variable on the other with a two-year time lag. Each path in the models was adjusted for age, gender, education, civil status, and children living at home. Indicators of the latent variable leadership were allowed to correlate between waves. The same bivariate models were used in order to test associations between managerial leadership and psychological demands, between decision authority and workplace violence (putative mediators) as well as between putative mediators, as described above and sickness absence (outcome). The models were adjusted for the same set of covariates as above. If there were significant paths between the predictor and the mediator and between the mediator and the outcome, a mediation model under the MSEM framework could be fitted. The second step was to apply such a model to our data. A longitudinal mediation model within an MSEM framework, in which leadership was measured at t-2 (in the years 2010 or 2012), psychological demands, decision authority and violence at t-1 (in the years 2012 or 2014) and sickness absence at t (in the years 2014 or 2016), was fitted. The model was adjusted for the same set of covariates as in the bivariate models. Such a model makes it possible to estimate the direct effect (the part of the exposure effect which was not mediated through psychological demands, decision authority, or violence) as well as the indirect effect (the part of the exposure effect which was mediated through psychological demands, decision authority or violence) between leadership and sickness absence.

In a third step, in order to examine if managerial leadership moderates the association between psychosocial work stressors (psychological demands, decision authority, or workplace violence) and sickness absence, we utilised bivariate models described in step 1 in a stratified model by managerial leadership. To determine whether the cross-lagged paths differed between good and bad leadership we conducted multiple-group analyses testing differences in each hypothesised and reverse association separately. We created two groups based on leadership (using the median value as a cut-off), then compared a model in which the paths were allowed to vary freely with a model in which the paths were constrained to be equal between good and bad leadership. The likelihood ratio test was used for comparing restricted and non-restricted models. A significant change in chi-square (df) between the non-restricted model and the restricted one indicates a poorer fit for the restricted model. The multilevel SEM models were built in MPLUS 7. All variables were treated as time-varying. Standardised estimates were reported for the final models. The fit statistics chi-square (df), the root mean square error of approximation (RMSEA), and the standardised root mean square residual (SRMR) were considered. Model fit is assumed to be acceptable when RMSEA ≤ 0.08 and SRMR ≤ 0.08 [43].

## Results

### Descriptive statistics

Means, standard deviations (or n) and percentages of the study variables for 2010, according to good/ poor leadership are presented separately in Tables 1 and 2.

**Table 1:** Means, standard deviations or n and percentages of the study variables for 2010

	n/ means	% / st.dev
Managerial Leadership (poor)	1.94	0.63
Psychological Demands (high)	2.66	0.55
Decision Authority (low)	2.05	0.72
Workplace violence		
No	970	79.12
Yes	256	20.88
Sickness Absence (register-based)		
0 days	2034	87.22
>0 days	298	12.78
Education (check description)		
1<= 9 years	175	13.49
2 Upper secondary school (2 years)	235	18.12
3 Upper secondary school (3–4 years)	186	14.34
4 University <3 years	198	15.27
5 University >= 3 years	503	38.78
Marital Status		
0 Married / cohabited	998	78.40
1 Not married / cohabited	275	21.60
Children living at home		
No	614	48.19
Yes	660	51.81

**Table 2:** Means, standard deviations or n and percentages of the study variables for 2010, separately according to poor/ good leadership of closest manager (the cut-off is the median value)

Poor leadership LD > 1.88 (median)	n/ means	% / st.dev
Psychological Demands (high)	614/2.76	0.52
Decision Authority (low)	614/2.17	0.71
Workplace violence		
No	526	76.12
Yes	165	23.88
Sickness Absence (register-based)		
0 days	602	86.49
>0 days	94	13.51
**Good leadership LD < 1.88 (median)**	**n/means**	**% / st.dev**
Psychological Demands (high)	548/2.53	0.56
Decision Authority (low)	548/1.93	0.67
Workplace violence		
No	391	83.19
Yes	79	16.81
Sickness Absence (register-based)		
0 days	418	88.56
>0 days	54	11.44

### Bivariate associations between managerial leadership and sickness absence

**Fig. 1:**
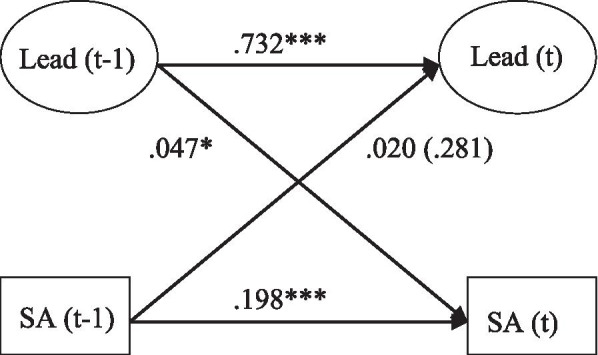
Relationship between poor leadership (Lead) and sickness absence (SA), adjusted for age, gender, education, civil status and children; RMSEA 0.036, SRMR 0.051

Figure 1 shows that the bivariate association between managerial leadership (t-1) and sickness absence (t) was 0.047 (*p=*0.011). Poorer perceived managerial leadership was thus associated with a higher risk of sickness absence two years later. The reversed path (from sickness absence (t-1) to managerial leadership (t)) was not statistically significant.

### Bivariate associations between managerial leadership and psychosocial work stressors

**Fig. 2:**
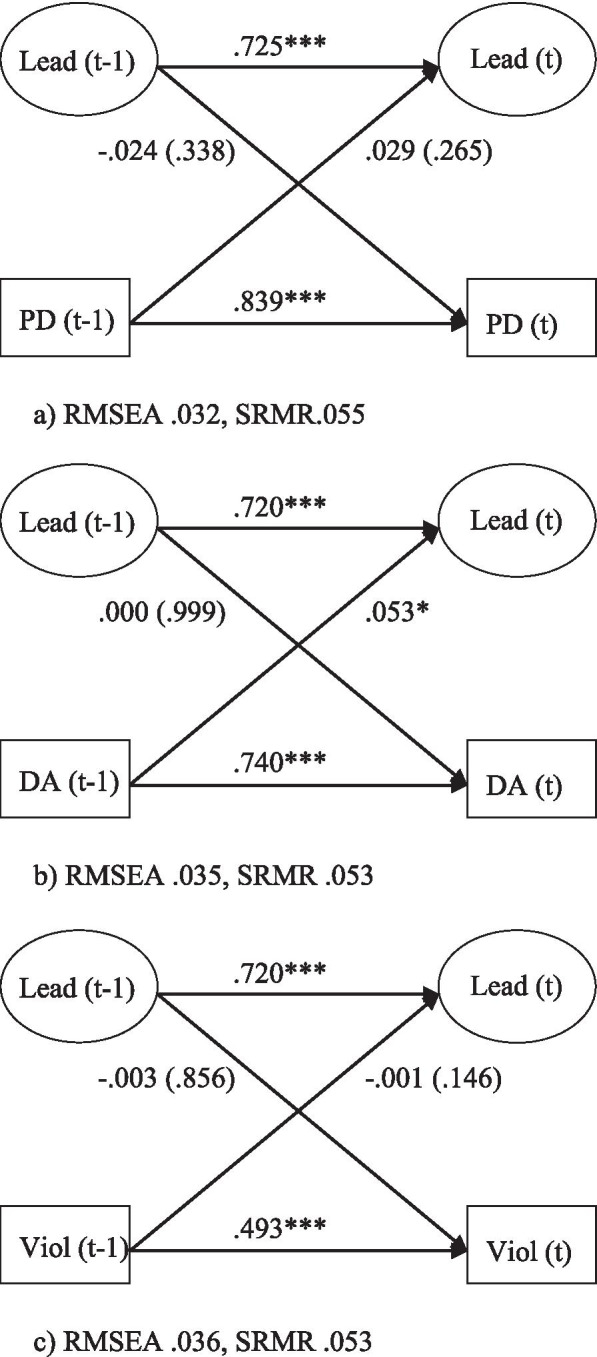
Relationship between poor leadership (Lead) and (**a**) psychological demands (PD), (**b**) poor decision authority (DA), and, (**c**) workplace violence (Viol), adjusted for age, gender, education, civil status and children

The bivariate associations between managerial leadership and psychological demands, decision authority and workplace violence are presented in Fig. 2a–c. We found no significant associations between managerial leadership (t-1) and psychosocial work stressors (t). One reverse association—between low decision authority and managerial leadership—was statistically significant (0.053, *p=*0.045).

### Bivariate associations between psychosocial work stressors and sickness absence

**Fig. 3:**
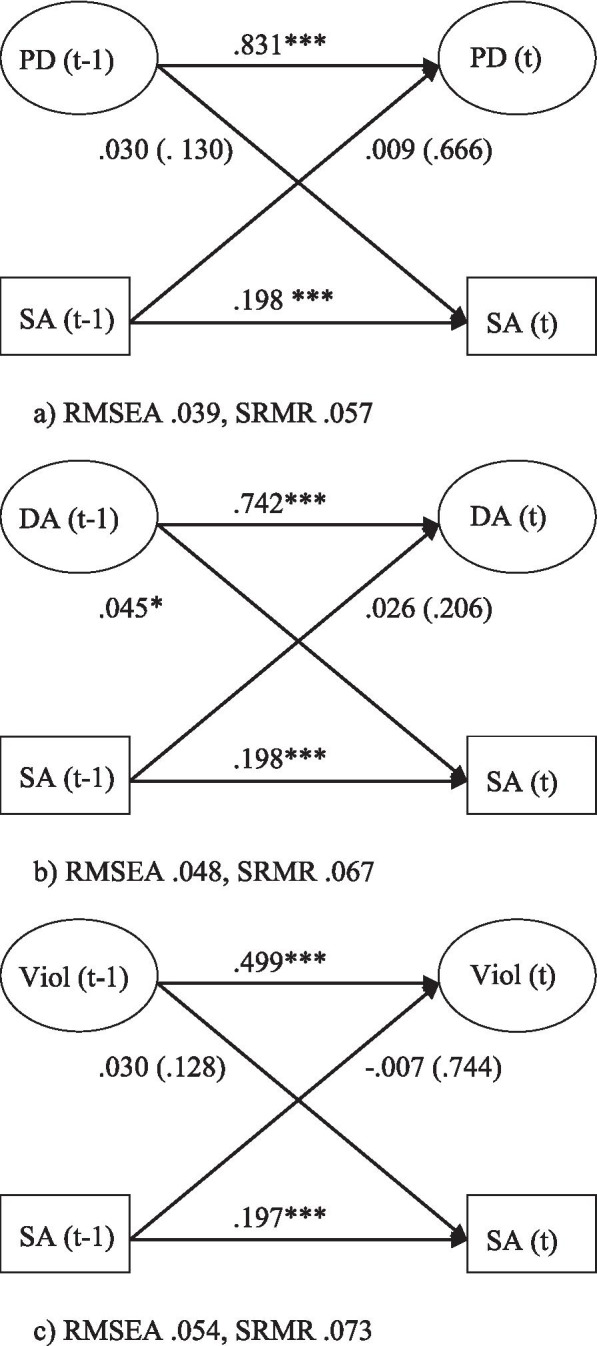
Relationship between (**a**) psychological demands (PD), (**b**) poor decision authority (DA), and, (**c**) workplace violence (Viol) and sickness absence (SA), adjusted for age, gender, education, civil status and children

The bivariate associations between psychosocial work stressors and sickness absence are presented in Fig. 3a–c. The estimate for the association between low decision authority (t-1) and sickness absence was 0.045 (*p=*0.032). Psychological demands and workplace violence (t-1) were not found to significantly predict sickness absence (t). Finally, no reversed paths were statistically significant.

### The moderating role of managerial leadership in the association between psychosocial work stressors and sickness absence

**Fig. 4:**
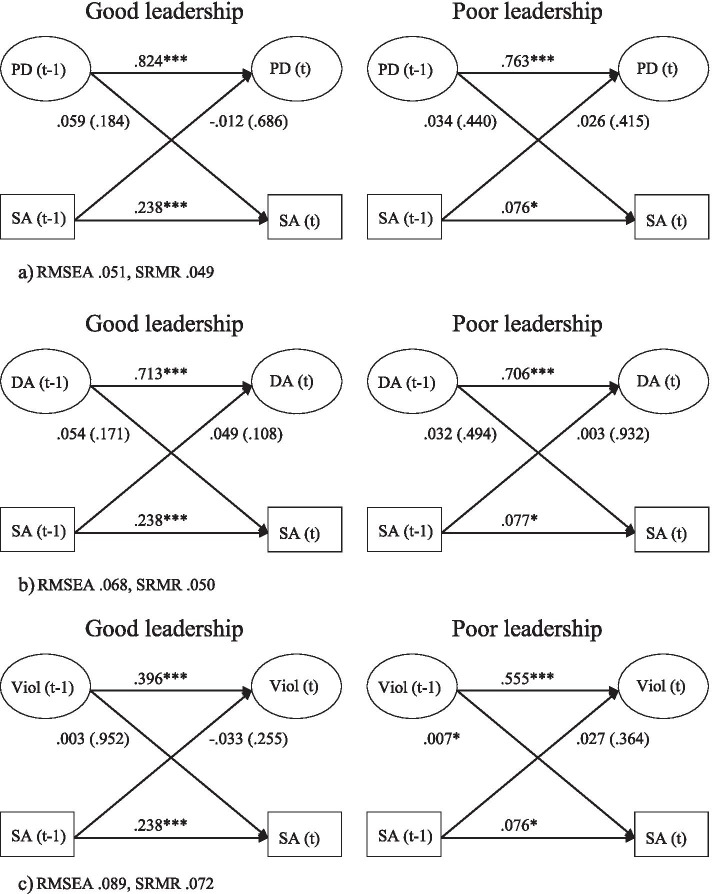
Models with leadership (good/ poor) as a moderator. Relationship between (**a**) psychological demands (PD), (**b**) poor decision authority (DA), and, (**c**) workplace violence (Viol) and sickness absence (SA), adjusted for age, gender, education, civil status and children

The bivariate associations between psychosocial work stressors and sickness absence are presented separately for those with good managerial leadership and poor managerial leadership in Fig. 4a-b. The paths between psychological demands (t-1) and sickness absence (t) and the paths between decision authority (t-1) and sickness absence (t) were not statistically significant. For workplace violence (Fig. 4c), significant associations were found for those with poor leadership (estimate: 0.007, *p=*0.041), but not for those with good leadership (estimate: 0.003, *p=* 0.952). The model in which the paths between workplace violence and sickness absence were constrained to be equal between good and poor leadership showed a poorer fit to the data than the model presented above where the paths were allowed to vary freely between good and poor leadership.

## Discussion

The aim of the present study was to examine the role of managerial leadership in the association between psychosocial work stressors (high psychological demands, low decision authority and exposure to workplace violence) and sickness absence among health and social care workers. We found support for the hypothesis that poor perceived leadership of the closest manager was associated with a higher risk of sickness absence over time, and that good managerial leadership buffered the effect of workplace violence on sickness absence. However, we could not find any support for the hypothesis that psychosocial work stressors mediate the association between poor managerial leadership and register-based sickness absence.

The finding that poor leadership of the closest manager was associated with higher risk for sickness absence two years later supports our first hypothesis and is in line with earlier studies [18]. The finding of the present study strengthens the evidence for an association between leadership and sickness absence by the fact that the reversed association—from sickness absence to managerial leadership—was controlled for, but showed no significant association, and that we used a register-based measure of sickness absence.

However, our second hypothesis was not confirmed. The association between poor perceived leadership of the closest superior and sickness absence was not mediated by high psychological demands, low decision authority or exposure to workplace violence. More specifically, even though there was a statistically significant association between low decision authority and sickness absence over time, managerial leadership was not associated with decision authority. Interestingly, we found a statistically significant association in the reverse direction from decision authority to managerial leadership, such that lower decision authority was related to poorer managerial leadership two years later. This may indicate that the employees’ perceptions of their managers’ leadership skills may be affected by the levels of decision authority they have been given regarding their work tasks over time.

With regards to psychological demands and workplace violence, no significant associations were found with either managerial leadership or sickness absence over time. These findings are not in line with two cross-sectional studies on nurses that did find significant associations between the closest managers’ leadership and decision authority [29, 44] and psychological demands [29] (to be noted Malloy and Penprase [44] did not support the latter association). One plausible reason for the inconsistency may be that these earlier studies were cross-sectional whereas the present study had a more robust research design, using a prospective approach with variables being measured two years apart, controlling for cross-sectional correlations and reversed longitudinal associations (i.e., from psychosocial work stressors to managerial leadership). Perhaps any influence of managerial leadership on psychosocial work stressors plays out rather contemporarily (our results also support cross-sectional relationships); hence, a two-year time span may be too long, and might fail to capture the mechanisms in question. In line with such a claim is, for instance, a study by Nielsen, Randall [30] where a mediating mechanism of work characteristics could be found when measuring leadership behaviour and work characteristics contemporarily, but not with measures 18 months apart. Thus, there is a need for more research scrutinising the mechanism utilising different and shorter time intervals.

Concerning managerial leadership as a potential moderator in the associations between psychosocial work stressors and sickness absence, managerial leadership only seemed to matter in the association between workplace violence and sickness absence. Thus, partial support was found for our third hypothesis. More specifically, only for those reporting poor managerial leadership was there a small association between workplace violence and sickness absence over time, meaning that in cases of experiencing workplace violence, poor leadership may increase the risk for subsequent sickness absence. Put differently, good leadership may protect the worker from suboptimal health outcomes caused by workplace violence. The effect was rather small, but as violence from patients and clients is more present in the health and social care sectors compared to other sectors [2, 13, 14], it may be of practical significance. Earlier studies examining potential moderator effects of leadership on the associations between psychosocial work stressors and health outcomes are rare [32] and results are mixed. For instance one study—using nationally representative work environment studies from two Nordic countries—did not support a buffering effect of good leadership compared to poor leadership on the association between emotional job demands and antidepressant treatment [34], whereas another study found an interaction effect of job strain and supportive leadership on poor well-being ten years later [33]. However, the direction of the effect found in the latter study was unexpected as those who reported low job strain together with a lack of supportive leadership had poorer well-being compared to those with high job strain. As has been acknowledged by scholars, more research is warranted on the possible stress buffering effects of good leadership [32].

In the present study we utilised an assessment of managerial leadership, which rather broadly measures rudimentary leadership behaviour in the Swedish context [23]. Lack of such leadership has been acknowledged as a psychosocial stressor which increases the risk of suboptimal health [22, 23]. Although, this measure foremost was developed to scrutinize leadership behaviours that may decrease subordinates’ stress and unhealth, there are resemblances with other concepts of leadership styles and behaviours [22], originally developed to foremost increase performance [25]. For example, a number of items pertain to task-oriented behaviours or to the contingent reward subscale of transactional leadership, which assesses to what extent the employee perceives that “the leader clarifies expectations and sets up constructive transactions for meeting these expectations” ([45], p 26). To a smaller extent, the present measurement also resembles relation-oriented behaviours and transformational leadership, where the latter during the last decades have dominated the leadership research field [32]. Finally, low values may pertain to more laissez-faire leadership behaviours (the absence of leadership). Many leadership scales have been found to correlate highly to each other. For instance, Zwingmann, Wegge [45] in a large multinational sample found that transformational leadership and contingent reward were highly correlated (about 0.90 on average), which indicates that these two often go hand in hand. Also, laisse-faire leadership showed strong negative correlations (-0.76 and -0.72) with both transformational leadership and contingent reward [45]. This implies that leaders usually do not lead by only adopting one style but by combining several. Nevertheless, the importance of different leadership styles and their impact on certain job characteristics and on sickness absence may vary in different industries on the labour market. In future research, it would be valuable to replicate this study in particular work settings within the health and social care industry, but also testing other leadership scales.

### Strengths and limitations

The present study had several strengths; it was based on a sizeable longitudinal cohort study, and was largely representative of the Swedish working population. Four time points were utilised and all our models were cross-lagged panel data models (controlling for cross-sectional associations, autoregressive associations and for reversed associations). A major strength with this method is that it measures within-individual variance, hence many unmeasured confounding variables, such as personality, are taken into account. In addition, this method models the unobservable correlation between the endogenous variable equation and the outcome equation, accounting for endogeneity issues. However, we do not know whether the choice of two years between the measurement points is optimal. Perhaps, some mechanisms are faster or take longer to evolve.

The decision to exclude workers who had changed manager between two subsequent surveys was taken to ensure that changes in estimates were not due to this particular change. Sensitivity analyses indicated that a few additional associations between variables were statistically significant when allowing for a change of manager between two waves. The present results may thus be considered conservative.

Sickness absence was register-based, in contrast to the predictor and mediator variables which were self-reported, thereby decreasing the risk for common-methods bias [46, 47]. Also, the measure should be accurate as it is linked to benefits for the individual. Another strength of the use of doctor-certified sickness absences 15 days or more is that it is likely to be associated with stress-related mental health problems, such as burnout and depression, that are common among health and social care workers. On the other hand, we lack information on shorter absence (1–14 days), which means that in the comparison group (0 days) there may be individuals who have many sickness absence days (spread over one year) that are never registered. Thus, the reported associations between our studied factors and sickness absence are likely to represent underestimations of the true situation and the results cannot be generalized to capture shorter (repeated) sick leaves, since for those partly different processes and incentives may be involved [11].

Whereas the large representative sample of Swedish health and social care workers is one of the major strengths of the present study, the heterogeneity also poses a potential drawback as the manager–employee relationship might differ between professions. For example, medical doctors, psychologists, nurses, and care assistants work under different conditions, and may have different expectations on their closest manager. The present study can be generalised to health-care professionals in general, but not claim to add knowledge about differences in the manager–employee relationship between professionals. Also, it is well known that leadership is not shouldered by formal managers alone, but often shared between formal leaders and other individuals in workgroups. The present study does not measure the more complex process of workplace leadership, but is restricted to the manager–employee relationship, as assessed by the employee. Moreover, although the managerial leadership scale measures a wide range of behaviours, it is not primarily developed to examine how managers best prevent violence from patients and clients.

With regard to workplace violence—as in many previous studies—it was measured by a single-item, which did not specify the perpetrator (although violence from patients and clients is the most common in these industries) or the severity of the violence. Furthermore, we do not know if there are psychological and sexual components to the workplace violence measured here. These issues correspond to shortcomings in the workplace violence literature in general [14].

## Conclusions

The leadership of the closest manager appears to be associated with sickness absence among health and social care workers, both directly and by buffering against negative health effects of certain work exposures. It does not, however, appear as if the closest manager has much influence on the psychosocial work stressors themselves. This indicates that, in the health and social care industry, the relationship between manager and subordinate is important for subordinates’ health, but that health and social care managers have limited influence on several of the work environment factors known to impact health outcomes. However, more research is warranted, for instance utilising shorter time intervals and different leadership scales and more specific measures of workplace violence.

## Supplementary Information

**Additional file 1.**

## Data Availability

The datasets generated and analysed during the current study are not publicly available due to legal restrictions. For data requests, please contact data manager Constanze Leineweber at contanze.leineweber@su.se.

## Abbreviations

DCSQ: Demand-Control-Support-Questionnaire
MSEM: multilevel structural equation modelling
SLOSH: Swedish Longitudinal Occupational Survey of Health
SEM: structural equation model
