# Supplementary material for: The role of managerial leadership in sickness absence in health and social care: antecedent or moderator in the association between psychosocial working conditions and register-based sickness absence? A longitudinal study based on a swedish cohort
Source: BMC Public Health. 2021 Dec 4;21:2215. doi: 10.1186/s12889-021-12236-z (PMC8642919; doi:10.1186/s12889-021-12236-z)
Supplement: Supplementary file 1 — Additional file 1. [file 12889_2021_12236_MOESM1_ESM.docx]

**Supplementary material**

Table S1. Descriptive and Pearson’s correlation for items of Managerial leadership scale*, measured at the first wave (2010).

| item | 1 | 2 | 3 | 4 | 5 | 6 | 7 | 8 | N | Mean | St. Dev. |
| --- | --- | --- | --- | --- | --- | --- | --- | --- | --- | --- | --- |
| 1 | – |  |  |  |  |  |  |  | 1,189 | 1.66 | .69 |
| 2 | .54 | – |  |  |  |  |  |  | 1,180 | 2.01 | .80 |
| 3 | .61 | .59 | – |  |  |  |  |  | 1,186 | 1.98 | .86 |
| 4 | .51 | .47 | .62 | – |  |  |  |  | 1,185 | 1.61 | .75 |
| 5 | .57 | .47 | .57 | .50 | – |  |  |  | 1,187 | 2.00 | .92 |
| 6 | .33 | .26 | .34 | .43 | .37 | – |  |  | 1,183 | 1.57 | .66 |
| 7 | .53 | .58 | .61 | .48 | .71 | .39 | – |  | 1,187 | 2.24 | .85 |
| 8 | .52 | .49 | .59 | .49 | .68 | .33 | .72 | – | 1,185 | 2.19 | .95 |
| 9 | .51 | .44 | .54 | .48 | .72 | .31 | .63 | .70 | 1,184 | 2.22 | .90 |

* Managerial leadership scale (Setterlind and Larsson 1995)

1. My boss gives me the information I need.

2. My boss is good at pushing through and carrying out changes.

3. My boss explains goals and sub-goals for our work so that I understand what they mean for my particular part of the work

4. I have a clear picture of what my boss expects of me.

5. My boss shows that he/she cares how things are for me and how I feel.

6. I have sufficient power in relation to my responsibilities.

7. My boss takes the time to become involved in his/her employees’ professional development.

8. My boss encourages my participation in the scheduling of my work.

9. I am praised by my boss if I have done something good.

(Note one item excluded: “*I receive criticism from my boss if I have done something that is not good.)*

**References**

Setterlind S, Larsson G (1995) The stress profile: a psychosocial approach to measuring stress Stress Medicine 11:85-92
